# Experimental data on the splitting tensile strength of bamboo reinforced lateritic concrete using different culm sizes

**DOI:** 10.1016/j.dib.2018.09.064

**Published:** 2018-09-27

**Authors:** Olumoyewa Dotun Atoyebi, Samson O. Odeyemi, Joy A. Orama

**Affiliations:** a Department of Civil Engineering, Landmark University, Omu-aran, Kwara State, Nigeria; b Department of Civil Engineering, Kwara State University, Malete, Kwara State, Nigeria

## Abstract

In this data article, the splitting tensile strengths of bamboo reinforced lateritic concrete with varying bamboo culm sizes are presented and compared with bamboo reinforced pure concrete (i.e. no lateritic replacement for fine aggregate). 25% of fine aggregate was replaced with laterite and concrete cylinders were cast using bamboo reinforcements both full culm sizes and half culm sizes. The cylinders of 300 mm height and 150 mm diameter were cured for 28 days and subjected to splitting tensile load at 7 days, 14 days and 28 days. Three samples were tested for each conditions and the average value computed.

**Specifications table**

**Table:** 

Subject area	*Civil Engineering*
More specific subject area	*Construction Materials, Waste Management*
Type of data	*Table, figure*
How data was acquired	*Casting concrete samples in the laboratory and applying splitting tensile load.*
Data format	*Raw*
Experimental factors	*Bamboo was coated with a mixture of bitumen and sharp sand, during the curing process; the concrete cylinders were stored in water to reduce shrinkage.*
Experimental features	*Fine aggregate replaced with lateritic soil to cast concrete cylinders reinforced with bamboo.*
Data source location	*Landmark University Concrete and Geotechnical Laboratory, Omu-aran, Kwara State. Nigeria.*
Data accessibility	*Data are as presented in this article*

**Value of the data**

• The test data allows for investigation on the use of bamboo as another possible material [1] for reinforcement in concrete.
• The data allows for the assessment of the possibility of replacing fine aggregate with laterite [2].
• The data presented can be used to examine bamboo culm sizes in the strength of concrete.

## 1 Data

The data presented information on splitting tensile strength of pure concrete and lateritic concrete reinforced with bamboo. Failure load of reinforced lateritic concrete cylinders with full culm and half culm bamboo reinforcement at 7, 14 and 28 days of curing were given. Splitting Tensile strength of pure concrete and lateritic concrete cylinders with full culm bamboo reinforcement at 7, 14 and 28 days of curing.

## 2 Experimental design, materials and methods

**Fig. 1:**
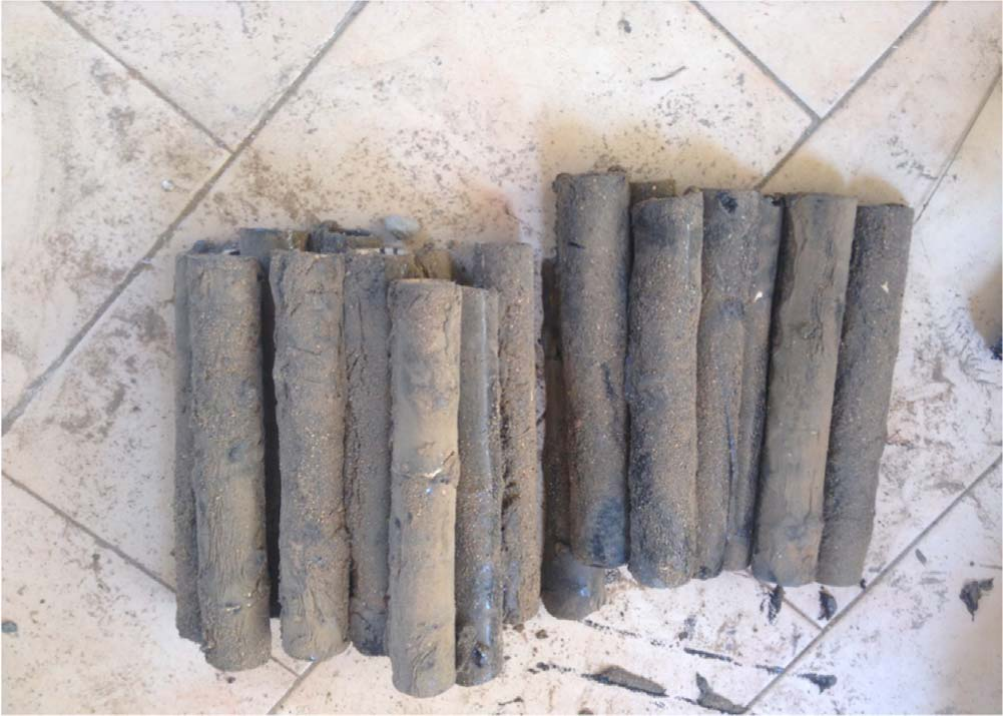
Full culm bamboo coated with bitumen and sand.

The aggregate materials and cement used for this research were collected from different locations in Omu-aran, Kwara State, Nigeria (8.1402°N, 5.0963°E). The bamboos which were also gotten from Landmark University farms were cut into the length needed some in full culm and others sliced vertically into half culms. Properties of the bamboo were as presented in Table 1, the bamboo were coated with bitumen mixed with sand (Fig. 1), this is to prevent the bamboo from soaking water in the concrete [3], 25% of fine aggregate was replaced with laterite. Concrete cylinders of 150 mm diameter and 300 mm height were cast and cured. Splitting tensile test was carried out on three (3) different specimens each at 7 days, 14 days and 28 days. Tables 2 and 3 shows the splitting tensile strength values at 7 days for full culm and half culm bamboo reinforcement respectively, the splitting tensile strength at 14 days for full culm and half culm bamboo reinforcement were presented in Tables 4 and 5 respectively while Tables 6 and 7 shows the strength values for 28 days. Table 8, Table 9, Table 10 shows the splitting tensile strength values at 7 days, 14 days and 28 days respectively for full culm bamboo reinforcement in pure concrete (Fig. 1).

**Table 1:** Some specific properties of bamboo.

Properties	Values
Specific gravity	0.575–0.655
Average weight	0.625 kg/m
Modulus of rupture	610–1600 kg/cm^2^
Modulus of elasticity	1.5–2.0 × 105 kg/cm^2^
Ultimate compressive stress	794–864 kg/cm^2^
Safe working stress in compression	105 kg/cm^2^
Safe working stress in tension	160–350 kg/cm^2^
Safe working stress in shear	115–180 kg/cm^2^
Bond stress	5.6 kg/cm^2^

**Table 2:** Splitting tensile strength at 7 days for full culm bamboo reinforcement in lateritic concrete.

Specimen no.	Max. load (KN)	Splitting strength (N/mm^2^)
F_1–7_	24.1	0.34
F_2–7_	29.7	0.42
F_3–7_	33.2	0. 47
Mean splitting tensile strength		0.38

**Table 3:** Splitting tensile strength at 7 days for half culm bamboo reinforcement in lateritic concrete.

Specimen no.	Max. load (KN)	Splitting strength (N/mm^2^)
H_1–7_	30	0.42
H_2–7_	18	0.54
H_3–7_	41	0.58
Mean splitting tensile strength		0.51

**Table 4:** Splitting tensile strength at 14 days for full culm bamboo reinforcement in lateritic concrete.

Specimen no.	Max. load (KN)	Splitting strength (N/mm^2^)
F_1–14_	50.1	0.71
F_2–14_	50.5	0.714
F_3–14_	51.7	0.73
Mean splitting tensile strength		0.72

**Table 5:** Splitting tensile strength at 14 days for half culm bamboo reinforcement in lateritic concrete.

Specimen no.	Max. load (KN)	Splitting strength (N/mm^2^)
H_1–14_	42	0.59
H_2–14_	78	1.1
H_3–14_	92	1.3
Mean splitting tensile strength		1.00

**Table 6:** Splitting tensile strength at 28 days for full culm bamboo reinforcement in lateritic concrete.

Specimen no.	Max. load (KN)	Splitting strength (N/mm^2^)
F_1–28_	55	0.78
F_2–28_	88	1.24
F_3–28_	41	0.58
Mean splitting tensile strength		0.87

**Table 7:** Splitting tensile strength at 28 days for half culm bamboo reinforcement in lateritic concrete.

Specimen no.	Max. load (KN)	Splitting strength (N/mm^2^)
H_1–28_	122	1.73
H_2–28_	43	0.61
H_3–28_	97	1.37
Mean splitting tensile strength		1.24

**Table 8:** Splitting tensile strength at 7 days for full culm bamboo reinforcement in pure concrete.

Specimen no.	Max. load (KN)	Splitting strength (N/mm^2^)
P_1–7_	26.3	0.37
P_2–7_	26.9	0.38
P_3–7_	28.2	0.40
Mean splitting tensile strength		0.375

**Table 9:** Splitting tensile strength at 14 days for full culm bamboo reinforcement in pure concrete.

Specimen no.	Max. load (KN)	Splitting strength (N/mm^2^)
P_1–14_	50.2	0.71
P_2–14_	52.1	0.74
P_3–14_	51.7	0.73
Mean splitting tensile strength		0.73

**Table 10:** Splitting tensile strength at 28 days for full culm bamboo reinforcement in pure concrete.

Specimen no.	Max. load (KN)	Splitting strength (N/mm^2^)
P_1–28_	87.1	1.23
P_2–28_	88.7	1.25
P_3–28_	91.2	1.29
Mean splitting tensile strength		1.26
