# Supplementary material for: Experimental data on the splitting tensile strength of bamboo reinforced lateritic concrete using different culm sizes
Source: Data Brief. 2018 Sep 27;20:1960–4. doi: 10.1016/j.dib.2018.09.064 (PMC6171081; doi:10.1016/j.dib.2018.09.064)
Supplement: Supplementary file 1 — Supplementary material [file mmc1.docx]

Declaration of Interest

This research work is part of a BSc Thesis of Orama Joy Ayebadogiye and there is no specific grant from funding agencies in the public, commercial, or non profiting sectors.
